# Personal and perceptual factors associated with the use of electronic cigarettes among university students in northern Thailand

**DOI:** 10.18332/tid/133640

**Published:** 2021-04-22

**Authors:** Chakkraphan Phetphum, Atchara Prajongjeep, Kanyarat Thawatchaijareonying, Thanchanok Wongwuttiyan, Mintra Wongjamnong, Somreuthai Yossuwan, Dueanchai Surapon

**Affiliations:** 1 Department of Community Health, Faculty of Public Health, Naresuan University, Phitsanulok, Thailand; 2 Tobacco Control Research Unit, Naresuan University, Phitsanulok, Thailand; 3 Department of Community Public Health, Sirindhorn College of Public Health, Phitsanulok, Thailand

**Keywords:** electronic cigarettes, smoking, university, students, Thailand

## Abstract

**INTRODUCTION:**

Although Thailand has banned the import of electronic cigarettes (e-cigarettes) into the kingdom, a range of evidence has revealed the widespread use of these illegal products, especially among university students. Too little is known about personal and perceptual factors for such behavior. This study aimed to investigate the prevalence and factors associated with the use of e-cigarettes among university students in Northern Thailand

**METHODS:**

A cross-sectional study was conducted with 792 university students in Northern Thailand between June and July 2020. An anonymous, self-administered questionnaire was implemented to collect relevant information. Bivariate and multivariate logistic regression analyses were applied to identify factors associated with the use of e-cigarettes in the last 30 days.

**RESULTS:**

The study revealed that 18.1% of the participants used e-cigarettes in the past 30 days. The personal factors associated with e-cigarette use among Thai university students included: studying in non-health related faculties (AOR=11.21; 95% CI: 4.88–25.71); having a friend using e-cigarettes (AOR=10.48; 95% CI: 5.96–18.41); having a lower GPA than the mean (AOR=1.93; 95% CI: 1.14– 3.28); and having a monthly income higher than the mean (AOR=1.74; 95% CI: 1.09–2.78). Regarding perceptual factors, there was a significant link between e-cigarette use and the perception that these modern cigarettes are less harmful than conventional cigarettes (AOR=2.47; 95% CI: 1.50–4.07), and perception that using e-cigarettes in public is not illegal (AOR=1.93; 95% CI: 1.19–3.15).

**CONCLUSIONS:**

E-cigarette use is widespread among Thai university students. Maintaining the enforcement policy on banning the importation of e-cigarettes into the country is thus needed. Also, further communication and public relations on the risks and legal prohibitions of e-cigarette use are needed among university students in the Northern Thailand.

## INTRODUCTION

Electronic cigarettes (e-cigarettes), also known as electronic nicotine delivery systems (ENDs), work by using heat to vaporize the e-liquid solution inside the cartridge, allowing the vapor to be inhaled. Important ingredients contained in the e-liquid solution generally consist of nicotine, propylene glycol, glycerin, flavoring agents, and other chemical substances^1^.

While there is no consensus on whether e-cigarettes are safer than conventional cigarettes and help quit smoking, these devices have had a dramatic increase in popularity and users worldwide^2^, especially among youths. In the United States, a rapid growth in e-cigarette use prevalence among high school students was observed, from 1.5% in 2011 to 20.8% in 2018^3^. Such a rise is expected as e-cigarettes are advertised as safe products, are inexpensive, are used for tobacco harm reduction and smoking cessation. They involve marketing strategies and used online media (e.g. websites and online communities) to provide information about prices and flavors, introduce new products to attract teenagers, demonstrate their use, and access as well as trade over the internet^4-6^.

Currently, 41 countries prohibit the sale, advertisement, and marketing of e-cigarettes^7^. Thailand is also one of these that bans the products by applying laws in four ways: 1) prohibition of imports into the kingdom, 2) prohibition of sale on websites or online networks, 3) prohibition of their use in non-smoking areas, and 4) prohibition of possession as an offence under the law. Although Thailand has strict legal measures against e-cigarettes, evidence has confirmed the spread of e-cigarette use in this country, especially among late teenagers, who are students in tertiary education and have more freedom to make their own decisions. The survey results of the Tobacco Control Research and Knowledge Management Center^8^ found that most university students knew of e-cigarettes, and more than one-third wanted to try them as they believed that these modern cigarettes are safer than the conventional ones, help lower the risk of being afflicted by lung cancers, and help to quit smoking. Additionally, Piyawan Boonphen et al.^9^ reported that 20.81% of Thai university students used e-cigarettes, and 26.70% switched between e-cigarettes and conventional cigarettes. The majority of these students had low legal knowledge, had positive attitudes toward e-cigarettes, had a close role-model who used e-cigarettes and were aware of e-cigarette advertising and marketing.

A wide range of previous studies has indicated several factors correlated with the use of e-cigarettes among youths. Most at-risk youths for using e-cigarettes were male^10^, were older teenagers who had: higher education^11^, better financial status^12^, a friend using e-cigarettes^13,14^, lower perceived dangers, and higher perceived benefits of e-cigarette use^15-24^. However, still little is known about the personal and perceptual factors for their use. Therefore, this study aimed to investigate the prevalence and factors associated with the use of e-cigarettes among university students in Northern Thailand.

## METHODS

### Participants and procedures

A cross-sectional study was conducted from June to July 2020 involving 3961 students who studied in the third year of a Bachelor’s program in 2020 at a university in Northern Thailand. This university consisted of 19 faculties, which could be categorized as faculties associated and not associated with health. The health-related Faculties consisted of: Dentistry, Nursing, Medicine, Pharmacy, Medical Sciences, Allied Health Sciences, and Public Health. Whereas non-health related faculties comprised: Agriculture, Natural Resources and Environment; Science; Engineering; Architecture; Social Sciences; Law; Humanities; Business Administration, Economics and Communication; Education; and International Colleges. This university had a policy of being a nonsmoking institution as required by law and conducted campaigns among students. However, much of the campaign content was still focused on traditional cigarettes, while the campaign content on the dangers and law related to electronic cigarettes was limited.

We calculated the sample size using the finite proportional population^25^ for the prevalence of the use of e-cigarettes among university students, with student population N=3961, the proportion of students who use e-cigarettes p=0.27^9^, delta calculated from 10% of p=0.027, alpha=0.05, and z(0.975)=1.96. A total of 824 participants were selected by a multi-stage sampling technique. Firstly, the faculties were drawn using stratified random sampling, and the sample size in each faculty was then calculated according to probability proportional to size. Secondly, the departments, representing each faculty, were selected by simple random sampling. Finally, systematic random sampling was used to recruit the participants using the list of the student ID numbers from each department as randomized number accounts, and set the sampling interval in each department using the calculation of the population (N) divided by the number of the sample groups (n). Inclusion criteria of this study were: 1) being university students, and 2) studying in the third year of a Bachelor’s degree. Exclusion criteria were: 1) being on leave and/ or not attending classes for three months, 2) being suspended by the university, 3) being unwilling to participate in the study, and 4) having a cognitive disorder due to diseases such as dementia, Parkinson’s disease or schizophrenia.

### Measurements

The instrument of this study was a self-administered questionnaire (Thai language, designed by the researchers) comprising both closed and open-ended questions, including the following three sections.

#### Section 1

Questionnaire on demographic information consisting of six questions on: 1) gender (male,female), 2) GPA of the current academic year, 3) monthly income, 4) current faculty (health-related, non-health related), 5) having any friends who use e-cigarettes (yes/ no), and 6) seeing e-cigarettes advertised online in the past year (yes/no). Questions 2 and 3 required a numeric short answer, while the rest were a checklist.

#### Section 2

Questionnaire assessing perceptions of e-cigarettes consisting of four questions: 1) e-cigarettes are less harmful than conventional cigarettes, 2) e-cigarettes help quit smoking conventional cigarettes, 3) e-cigarettes help improve appearance to be smarter and more modern, and 4) using e-cigarettes in public is not illegal. Levels of all perceptions were measured on a 5-point Likert scale (1=totally disagree, 2=disagree, 3=unsure, 4=agree, 5=totally agree).

#### Section 3

Questionnaire assessing the use of e-cigarettes consisting of six questions: 1) experience in using e-cigarettes in the past 30 days (yes/no); 2) frequency of e-cigarette use (every day, some days); 3) age (years) initiated e-cigarette use; 4) time most frequently using e-cigarettes (immediately after waking up, before/after meals, free/break time, when drinking alcohol, when visiting entertainment venues, other); 5) usage of e-cigarettes (using only e-cigarettes, switching between electronic and conventional cigarettes); and 6) intention to stop using e-cigarettes (yes/no). Question 3 required a numeric short answer and the rest were a checklist. Questions 2–6 were for those who used e-cigarettes in the past 30 days.

The questionnaire had passed a content validity check by three qualified experts and all questions had IOC (Item Objective Congruence Index) for each content >0.5^26^. The questionnaire was then pilot tested with 30 third-year students who were not in the sample. Cronbach coefficient alpha value for Section 2 from the pilot study was 0.73, which met the specified criterion of 0.7^27^.

### Data collection

In the data collection process, we met the participants from each faculty on the designated time and clarified the importance and objectives of the research. Information sheets and consent forms were distributed to all students, and an active consent was received from the participants. The respondents were duly informed that participation in the study was voluntary, and their identity would remain anonymous. After that, copies of the questionnaire were distributed to all participants to be answered within 60 min. After the due time, we collected the questionnaires immediately. We checked the data integrity and found that 792 questionnaires were completed with a response rate of 96.12%.

### Statistical analysis

Analyses were conducted using IBM SPSS Statistics version 17 for Windows. The characteristics of independent and dependent variables were analyzed using descriptive statistics, including frequency, percentage, mean and standard deviation. Meanwhile, multivariable logistic regression was conducted to assess factors associated with the use of e-cigarettes. Firstly, continuous variables were divided into two groups, following the mean of the sample (higher and lower than the mean). Two variables designated included monthly income (>mean and <mean; with mean=8647 Baht; 1000 Baht about US$32) and current GPA (>mean and <mean; with mean=3.09). Regarding perception variables, we imported the initial model in four questions (variables) and categorized each level of the perceptions into two groups (group 1: agree or totally agree, and group 2: unsure, disagree, or totally disagree).

After that, all variables were input into the initial model. Bivariate logistic regression analyses were applied using crude odds ratios (ORs) to assess the associations between the use of e-cigarettes and all independent variables (p<0.25). Besides, we used a multivariable logistic regression analysis for control of the variables by using adjusted odds ratios (AORs). Level of statistical significance was set at p<0.05 and the regression results were presented as crude odds ratios (OR) and adjusted odds ratios (AOR) with 95% confidence interval (CI). Finally, we tested the model’s goodness-of-fit using Hosmer-Lemeshow chi-squared test and found that the last model (step 6) had p=0.289 and was acceptable as the value was higher than 0.05.

## RESULTS

Of 792 respondents , 649 had never used an e-cigarette in the past 30 days (81.9%), while 143 were current users (18.1%). Most of the e-cigarette users used e-cigarettes sometimes (59.4%), used switching to conventional cigarettes (78.3%), and few used only e-cigarettes (21.7%). Most used e-cigarettes for the first time after entering the university (76.2%), while the others had used them since high school (23.8%). The time most frequently using e-cigarettes was when they had free time (40.6%), followed by when drinking alcohol (38.5%), immediately after waking up (11.9%), before/after having a meal (4.9%), and only when visiting entertainment venues (4.2%). Just over half of the users had intention to stop using e-cigarettes in the next 6 months (51.0%).

Majority of the e-cigarette users were male (73.4%), had a GPA lower than the mean (55.2%), were currently studying in non-health related faculties (94.4%) and had monthly income higher than the mean (58.7%). Most of them had friends using e-cigarettes (83.2%) and had seen e-cigarette advertisements on the internet in the past year (58.0%). More than half of the users had positive perception of e-cigarettes for all questions, especially for two items: 1) e-cigarettes are less harmful than conventional cigarettes, and 2) e-cigarettes help quit smoking conventional cigarettes. On these two items, e-cigarette users more strongly agreed than non-users. There was also an aspect where e-cigarette users and non-users had opinions in the same direction. These two groups strongly agreed that using e-cigarettes in public is not illegal. There is only one point that the e-cigarette users disagreed with rather than agreed, ‘e-cigarettes help improve appearance to be smarter and more up to date’. However, the number was still higher than among non-users (Table 1).

**Table 1:** Prevalence of e-cigarette use by personal and perceptual factors among Thai university students, 2020 (N=792)

*Personal and perceptual factors*	*All samples (n=792) n (%)*	*E-cig non-users (n=649) n (%)*	*E-cig users (n=143) n (%)*
**Gender**			
Female	367 (46.3)	329 (50.7)	38 (26.6)
Male	425 (53.7)	320 (49.3)	105 (73.4)
**GPA** (mean=3.09, SD=0.514)			
Higher than the mean	597 (75.4)	533 (82.1)	64 (44.8)
Lower than the mean	195 (24.6)	116 (17.9)	79 (55.2)
**Current faculty**			
Related to health	240 (30.3)	232 (35.7)	8 (5.6)
Non-health related	552 (69.7)	417 (64.3)	135 (94.4)
Monthly income* ( mean=8467, SD =3567)			
Lower than the mean	425 (53.7)	366 (43.6)	59 (41.3)
Higher than the mean	367 (46.3)	283 (56.4)	84 (58.7)
**Have a friend who uses e-cigarettes**			
No	451 (56.9)	427 (65.8)	24 (16.8)
Yes	341 (43.1)	222 (34.3)	119 (83.2)
**Have seen e-cigarette advertisements on the internet**			
No	413 (52.1)	353 (54.4)	60 (42.0)
Yes	379 (47.9)	296 (45.6)	83 (58.0)
**E-cigarettes are less harmful than conventional cigarettes**			
Unsure, disagree, and totally disagree	363 (45.8)	324 (49.9)	39 (27.3)
Agree, and totally agree	429 (54.2)	325 (50.1)	104 (72.7)
**E-cigarettes help quit smoking conventional cigarettes**			
Unsure, disagree, and totally disagree	426 (53.8)	362 (55.8)	64 (44.8)
Agree, and totally agree	366 (46.2)	287 (44.2)	79 (55.2)
**E-cigarettes help improve appearance to be smarter and more up to date**			
Unsure, disagree, and totally disagree	596 (75.3)	497 (76.6)	99 (69.2)
Agree, and totally agree	196 (24.7)	152 (23.4)	44 (30.8)
**Using an e-cigarette in public is not illegal**			
Unsure, disagree, and totally disagree	372 (47.0)	313 (48.2)	59 (41.3)
Agree, and totally agree	420 (53.0)	336 (51.8)	84 (58.7)

* Thai Baht (1000 Baht about US$32). E-cig: electronic cigarette.

The results of multivariate logistic regression analysis (Table 2) showed that six variables were statistically significantly associated with the use of e-cigarettes among the university students. These were four from personal variables and two from perceptual variables. The personal variables consisted of studying in non-health related faculties (AOR=11.21; 95% CI: 4.88–25.71, p<0.001), having a friend using e-cigarettes (AOR=10.48; 95% CI: 5.96–18.41, p<0.001), having a lower GPA than the mean (AOR=1.93; 95% CI: 1.14–3.28, p=0.014), and having a higher monthly income than the mean (AOR=1.74; 95% CI: 1.09–2.78, p=0.020). Perceptual variables included the perception that e-cigarettes are less harmful than conventional cigarettes (AOR=2.47; 95% CI: 1.50–4.07, p<0.001) and the perception that using e-cigarettes in public is not illegal (AOR=1.93; 95% CI: 1.19–3.15, p=0.008). The other five independent variables that were input into the multivariate logistic regression model showed no statistical significance, consisting of gender, have seen e-cigarette advertisements on the internet, the perception that e-cigarettes could help quit smoking conventional cigarettes, and the perception that e-cigarettes help improve appearance to be smarter and more up to date.

**Table 2:** Correlates of e-cigarette use among Thai university students, 2020 (N=792)

*Personal and perceptual factors*	*All samples (n=792) n (%)*	*E-cig users (n=143) n (%)*	*OR (95% CI)*	*AOR (95% CI)*
**Gender**				
Female (Ref.)	367 (46.3)	38 (10.4)	1.0	1.0
Male	425 (53.7)	105 (24.7)	2.84 (1.90–4.25)*	1.13 (0.61–2.10)
**GPA**				
Higher than the mean (Ref.)	597 (75.4)	64 (10.7)	1.0	1.0
Lower than the mean	195 (24.6)	79 (40.5)	5.67 (3.86–8.34)*	1.93 (1.14–3.28)**
**Current faculty**				
Related to health (Ref.)	240 (30.3)	8 (3.3)	1.0	1.0
Non-health related	552 (69.7)	135 (24.5)	9.39 (4.52–19.50)*	11.21 (4.88–25.71)**
**Monthly income**				
Lower than the mean (Ref.)	425 (53.7)	59 (13.9)	1.0	1.0
Higher than the mean	367 (46.3)	84 (22.9)	1.84 (1.28–2.66)*	1.74 (1.09–2.78)**
**Have a friend who uses e-cigarettes**				
No (Ref.)	451 (56.9)	24 (5.3)	1.0	1.0
Yes	341 (43.1)	119 (34.9)	9.54 (5.98–15.22)*	10.48 (5.96–18.41)**
**Have seen e-cigarette advertisements on the internet**				
No (Ref.)	413 (52.1)	60 (14.5)	1.0	1.0
Yes	379 (47.9)	83 (21.9)	1.65 (1.14–2.38)*	1.28 (0.83–2.00)
**E-cigarettes are less harmful than conventional cigarettes**				
Unsure, disagree, and totally disagree (Ref.)	363 (45.8)	39 (10.7)	1.0	1.0
Agree, and totally agree	429 (54.2)	104 (24.2)	2.66 (1.78–3.96)*	2.47 (1.50–4.07)**
**E-cigarettes help quit smoking conventional cigarettes**				
Unsure, disagree, and totally disagree (Ref.)	426 (53.8)	64 (15.0)	1.0	1.0
Agree, and totally agree	366 (46.2)	79 (21.6)	1.56 (1.08–2.24)*	1.41 (0.83–2.41)
**E-cigarettes help improve appearance to be smarter and more up to date**				
Unsure, disagree, and totally disagree (Ref.)	596 (75.3)	99 (16.6)	1.0	1.0
Agree, and totally agree	196 (24.7)	44 (22.4)	1.45 (0.98–2.17)*	1.06 (0.58–1.95)
**Using an e-cigarette in public is not illegal**				
Unsure, disagree, and totally disagree (Ref.)	372 (47.0)	59 (15.9)	1.0	1.0
Agree, and totally agree	420 (53.0)	84 (20.0)	1.33 (0.92–1.91)*	1.932 (1.19–3.15)**

AOR: adjusted odds ratio. Ref.: reference group. E-cig: electronic cigarette.

* p<0.25,

** p<0.05.

## DISCUSSION

Importation and possession of e-cigarettes in Thailand are illegal. However, smuggling importation through complicated illicit mechanisms are extensive, and the law enforcement is not sufficiently strict to impose punishment for e-cigarette sellers and users in the country; therefore, use of these products can be seen frequently^8^, especially among students. This current research found that 18.1% of university students in the Northern Thailand used e-cigarettes, which is consistent with the previous survey on e-cigarette use among university students in Bangkok (the capital of Thailand), reporting 21.0%^9^. This relatively high prevalence could be affected by marketing, advertising and trading of e-cigarettes undertaken on the internet without a geographical boundary^4-6^. Therefore, residents in any part of Thailand can access and possess e-cigarettes the same way. This statement could be confirmed by the results of this current research, which found that almost half of both users and non-users (47.9%) had seen online advertisements of e-cigarettes and a higher number (58.0%) is observed when focusing only on the group of e-cigarette users.

This study found that students who had friends who used e-cigarettes had almost 11 times the probability of using e-cigarettes as those who did not have such friends. This finding is consistent with previous research, which suggests that friends are the reference group for considering initiating e-cigarette use^13,14^. Students who had a higher monthly income than the mean were two times as likely to use the devices as students who had lower monthly income than the mean, consistent with prior research^12^. University students, also counted as late teenagers, obtain their monthly earnings from parents and/or part-time jobs, and so they are responsible for managing their own income. Money left from essential expenses allows them to spend on e-cigarettes. Moreover, it was also found that students studying in non-health related faculties and having a lower GPA than the mean had 11 times and 2 times, respectively, higher likelihood to use e-cigarettes compared to students who studied in health-related faculties such as medicine, nursing, and public health. This may be because health profession students are more aware of the risks and adverse health effects of e-cigarette use.

Perception refers to the process a person uses to interpret different external stimuli. An individual is likely to react to a stimulus favorably if they have a positive perception of it. The results of this research indicate that more than half of the university students perceived e-cigarettes more positively than negatively, based on two erroneous views; 54.2% agreed that e-cigarettes are less harmful than conventional cigarettes, and 53.0% agreed that using e-cigarettes in public is not illegal. Prior studies also revealed that youths who had a positive perception of e-cigarettes were more likely to use the devices than those who had negative perception^15-24^. The current research demonstrated that there is an association between e-cigarette use and the abovementioned two erroneous perceptions. Youths with low harm perceptions of e-cigarettes are less likely to be aware of vaping health risks such as heart attack and acute pneumonia, which can be fatal or cause long-term hospitalization. Likewise, youths who have uninformed perceptions regarding the prohibition of e-cigarette use in public places are likely to be unaware that such actions could result in fines and prosecution. In contrast, e-cigarettes were equally perceived as a smoking cessation aid by the users and non-users and such perception had no association with e-cigarette use among the students. This result is not surprising as young smokers who have not been exposed to the negative effects of smoking are unlikely to attempt to quit.

Considering lessons from the ineffective Thai law enforcement allowing underage youths to easily access conventional cigarettes through retail stores without any obstacles^28,29^, maintaining the ban on importation of e-cigarettes into the kingdom is still the best measure^1^. This strategy should be effective because it considers a precautionary measure from upstream. It could also be more practical than the underage sale restriction since it is likely to reduce barriers against law enforcement procedures. Also, it is expected that maintaining strict legislations will help to prolong the spread of positive social norms regarding e-cigarette use. Overall, this strategy could help to slow the epidemic of e-cigarette use among young people, and the increase rates should not be as rapid as are currently in many countries where e-cigarettes are legitimate^3,20^.

In addition, this research found that the university students still have wrong perceptions, especially that e-cigarettes are less risky than conventional cigarettes and the devices can be used in public areas in general without breaking the law. These findings urge the relevant government agencies to seek proactive communication schemes to improve the accuracy in perceptions on the dangers and adverse health effects of e-cigarettes. This strategy could be as important as communication and public relations for improving accurate perception on possession and use of e-cigarettes as illegal activities.

### Limitations

This research has several limitations. First, the samples were recruited from one university in Northern Thailand; therefore, a generalization of the findings is limited to border university students. Second, this research is a cross-sectional study; thus, it could not conclude on the causal relationships. Third, this research fails to achieve information about types of e-cigarettes and e-liquid solutions used and whether they contained nicotine. Future research should, therefore, study more representative samples to be able to generalize to the national level, consider conducting a longitudinal study, and investigating the types of e-cigarettes and the nicotine contained in e-liquids.

## CONCLUSIONS

E-cigarette use is widespread among Thai university students, especially among those who are studying in non-health related faculties, have a friend using e-cigarettes, low GPA, and a high monthly income, as well as mistaken perceptions on e-cigarettes. This study supports the importance of maintaining the policy on banning the importation of electronic cigarettes into the country. Additionally, further communication and public relations on the dangers and legal prohibitions of the use of e-cigarettes among Thai university students should be emphasized.
